# Epidemiological characteristics and risk factors of multidrug-resistant tuberculosis in Luoyang, China

**DOI:** 10.3389/fpubh.2023.1117101

**Published:** 2023-05-09

**Authors:** Zhenzhen Wang, Yi Hou, Tengfei Guo, Tao Jiang, Liang Xu, Hongxia Hu, Zhanqin Zhao, Yun Xue

**Affiliations:** ^1^The First Affiliated Hospital and Clinical Medical College, Henan University of Science and Technology, Luoyang, China; ^2^School of Medical Technology and Engineering, Henan University of Science and Technology, Luoyang, China; ^3^Luoyang Center for Disease Control and Prevention, Luoyang, China; ^4^Animal Science and Technology, Henan University of Science and Technology, Luoyang, China

**Keywords:** multidrug resistant tuberculosis (MDR-TB), transmission, risk factors, epidemiology, high-resolution melting curve (HRM)

## Abstract

**Objective:**

We aimed to examine the prevalence of multidrug-resistant tuberculosis (MDR-TB) in Luoyang, China, identify related risk factors, inform clinical practices, and establish standardized anti-tubercular treatment regimens.

**Methods:**

We conducted a retrospective analysis of high-resolution melting curve (HRM) data from 17,773 cases (2,748 of which were positive) between June 2019 and May 2022 to assess the prevalence of MDR-TB and to identify its associated risk factors.

**Results:**

Between June 2019 and May 2022, out of the 17,773 HRM results, 2,748 were HRM-positive, and 312 were MDR-TB cases. The detection rates for HRM-positive and MDR-TB were 17.0 and 12.1% for males, and 12.4 and 8.2% for females, respectively. The MDR-TB detection rate was higher in the urban areas (14.6%) than in the rural areas (10.6%) and more common among individuals under 51 years of age (14.1%) than those over 50 years of age (9.3%). Notably, the rate of detecting MDR-TB was 18.3% higher in new male patients than in new female patients, which was at 10.6%, and this difference was statistically significant (*p* < 0.001). Moreover, the rate of MDR detection in females who had received anti-tuberculosis treatment (21.3%) was higher than that in males (16.9%). In the multivariate model that considered the results of the sputum smear and detection time, MDR-TB was positively correlated with a history of tuberculosis (TB) treatment, being male, being younger than 51 years, and living in urban areas.

**Conclusion:**

Local TB infections are complex and diverse; therefore, more comprehensive monitoring methods are needed to curb the spread of MDR-TB.

## Introduction

MDR-TB is a pressing global issue in the field of anti-infective therapies (1). The worldwide burden of TB was substantial in 2019, with an estimated 10 million people infected with the disease and 1.45 million of them succumbing to TB-related complications. A total of 465,000 people were diagnosed with rifampicin-resistant TB (RR-TB), of which ~78% were categorized as MDR-TB in 2019 (2, 3). Among high-burden countries, China has a proportion of MDR-TB cases of ~4.5%, which surpasses the global average of 3.3%. (1). MDR patients limit the rational use of clinical resources, and this contributes to a higher mortality rate (4). Timely diagnosis, rapid detection of drug resistance, and effective treatment regimens are critical measures for the effective control of MDR (5, 6). HRM is a rapid molecular diagnostic technique commonly used in clinical settings that can be used for tuberculosis diagnosis and resistance molecular locus detection of first- and second-line anti-tuberculosis drugs (7). Faced with complex drug-resistant cases, we need more surveillance data to estimate the risk of transmission of drug-resistant TB. Therefore, having an understanding of the prevalence of drug-resistant tuberculosis (DR-TB) in this region can help optimize the utilization of limited resources to control the spread of TB (8–11). Based on the results of the HRM test, this study investigated the population characteristics of MDR-TB and the risk factors related to it in this area to provide epidemiological evidence for the management of clinical MDR-TB.

## Materials and methods

### Data analysis

From 1 June 2019 to 31 May 2022, we conducted a retrospective analysis of the HRM result records of a total of 17,773 cases at the First Affiliated Hospital of Henan University of Science and Technology. Finally, we included 2,843 positive HRM results in the study. In the study, we collected the relevant clinical information concerning these cases by consulting the infectious disease reporting network and the electronic medical record system of our hospital: (1) population information: gender and age; (2) other factors: tuberculosis treatment history, regional distribution, testing year, sputum smear results, HRM results, molecular drug sensitivity results, and mutation site to MDR-TB.

### Classification and definition

Patients who underwent HRM testing were categorized as follows:

(1) Patients who tested negative for sputum tuberculosis test (13,835 out of 17,773, 77.8%) but had a history of close contact with infectious tuberculosis exhibited corresponding clinical manifestations (such as cough, spitting, low-grade fever and night sweats (867 out of 17,773, 4.9%), or displayed abnormal shadows or active tuberculosis changes in imaging examinations with no improvement after 2–4 weeks of pneumonia treatment (156 out of 17,773, 0.8%); (2) patients with a history of tuberculosis treatment (previously cured or not completely cured) and tested negative for sputum coating (403 out of 17,773, 2.3%); (3) patients with tested positive for sputum smear (2,095 out of 17,773, 11.8%); (4) and children who tested positive for a strong tuberculin test (5 units, equivalent to 1: 2000) and exhibited clinical symptoms of tuberculosis (375 out of 17,773, 2.1%).

New patients: those who had never received anti-tubercular drugs, those who had taken less than a full course of standardized regular medication, and those who had received irregular treatment for < 1 month. Retreatment patients: patients who received irregular and inappropriate anti-tuberculosis treatment for at least 1 month due to tuberculosis, as well as those who had experienced initial treatment failure and relapse (12).

Regional distribution: urban area, Luoyang downtown (including six administrative districts); rural areas, surrounding counties and townships of Luoyang downtown (including the counties of Luanchuan, Yanshi, Ruyang, Mengjin, Xin'an, Songxian, Yiyang, Luoning, and Yichuan).

MDR-TB is a form of tuberculosis that is resistant to at least isoniazid (INH) and rifampicin (RFP). In this study, rifampicin resistance was identified by targeting an 81-bp fragment of the *rpoB* gene (codons 507–533) known as the rifampicin resistance determining region, RRDR). Our primer probes were designed to cover codon regions 507–512, 521–528, 513–520, and 529–533, respectively. The sites responsible for isoniazid resistance included the *katG* 315, the *inhA* region promoter (locus −17 ~ −8 locus), the *ahpC* promoter region (locus −44 ~ −30 and −15 ~ 3), and the *inhA* 94.

### Statistical analysis

HRM-positive cases were categorized according to sex, age, history of TB treatment, regional distribution, and smear results. The resistance pattern of the tuberculosis-infected population in this area was analyzed, and the MDR-TB cases were stratified by age to confirm the population characteristics. Duplicated participants were excluded before the analysis based on factors such as name, gender, and age. People under 15 and over 86 years of age were divided into separate groups, whereas the others were presented as one group every 5 years of age. Moreover, the Pearson chi-squared test was used to determine whether there was a significant statistical difference in the MDR rates between genders for different variables. Furthermore, a multivariate logistic regression model was created, including all variables that could potentially affect the development of MDR, to ascertain the relationship between these variables and the development of MDR. The analysis produced an adjusted odds ratio (OR) and their corresponding 95% confidence intervals (95% CI), with a *P* < 0.05 deemed statistically significant. All analyses were conducted using STATA/SE15.1 software (Stata Statistical Software: Release 15. 1 College Station, TX, USA).

## Results

**Figure 1:**
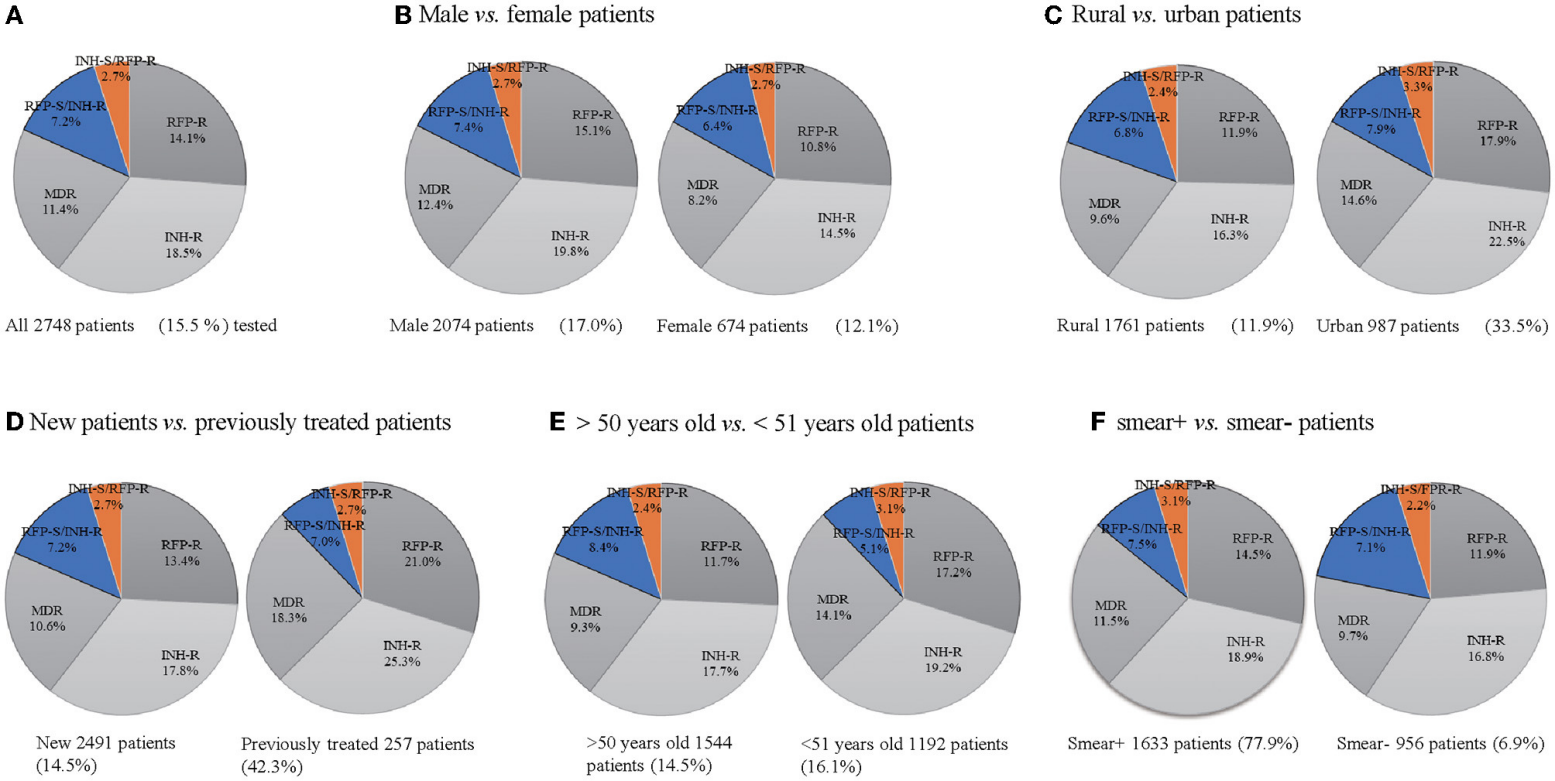
Drug resistance mode of TB cases **(A–F)**. Drug-resistant mode of all 2,748 TB patients tested **(A)**, 674 female and male patients **(B)**, 1,761 rural patients and 987 urban patients **(C)**, 257 previously treated TB patients and 2,491 new TB patients **(D)**, 1,544 TB patients of more than 50 years and 1,192 TB patients younger than 51 years **(E)**, 956 sputum smear-positive and 1,633 sputum smear-negative patients **(F)**. INH-R, INH-resistant; RIF-R, RIF-resistant; MDR, INH and RFP resistant; RFP-S/INH-R, RIF-susceptible and INH-resistant; INH-S/RFP-R, INH-susceptible and RIF-resistant.

From June 2019 to May 2022, a total of 17,773 patients underwent HRM testing. Of these, 15.5% (2,748) were HRM-positive patients, and 11.4% (312) were MDR-TB. The resistance patterns are shown in Figure 1, where 18.5% (509) were isoniazid resistant, 14.1% (387) were rifampicin-resistant, 7.2% (197) were resistant to isoniazid sensitive to rifampicin, 2.7% (75) were resistant to rifampicin sensitive to isoniazid, and 11.4% (54) were resistant to both isoniazid and rifampicin (Figure 1A). The HRM-positive rate was 17.0% (2,074) for males and 12.1% (674) for females, with the ratio of males to females being 3:1. MDR detection rates for males and females were 12.4% and 8.2%, respectively (Figure 1B). The positive rates of HRM in the urban and rural areas were 33.5% (987) and 16.9% (1,761), while the MDR detection rates were 14.6% and 9.6%, respectively (Figure 1C). The HRM positive results were significantly higher in the newly diagnosed population (2,591) than in the retreatment cases (257), but the HRM positive rate (42.3%) and the MDR detection rate (18.3%) of the retreatment cases were much higher than those of the first-time patients (14.5%, 10.6%) (Figure 1D). The HRM positive rate (16.1%) and MDR detection rate (14.1%) in patients younger than 51 years were higher than those older than 50 years (Figure 1E). The detection rates of HRM-positive and MDR in sputum-positive patients were 77.9 and 11.5%, while they were only 6.9 and 9.7% in the sputum-negative populations (Figure 1F).

During the study period, the MDR detection rate tended to decrease in both males and females (males 13.2–13.6– 0.6%, females 11.8–7.2–6.0%), with a male-to-female ratio of 4.7. In addition, we found that MDR detection was higher among newly diagnosed males (12.0%) than females (6.4%), whereas in patients who had received TB treatment, it was higher among females (21.3%) than males (16.9%). MDR-TB accounted for 16.1% of urban males and 10.1% of females, while it accounted for 10.3% of rural males and 7.0% of females. The MDR detection rate in patients < 51 years was 14.1% (males: 15.6%, females: 10.1%), which was higher than 9.3% in patients > 50 years (males: 10.2%, females: 6.2%). At the same time, the MDR detection rate of sputum-positive patients was 12.7% for males and 7.2% for females, while it was 10.4% for males and 8.0% for females in the sputum-negative group (Table 1).

**Table 1:** Detection of MDR-TB under different correlation variables by gender.

	**Gender**	
	**Males** ***n*** **(%)**	**Females** ***n*** **(%)**
HRM-Test-Result	12,217 (68.7)	5,556 (31.2)
MDR-TB	257 (12.4)	55 (8.2)
**Patient category**		
New cases	227 (12.0)	38 (6.4)
Previously treated cases	30 (16.9)	17 (21.3)
**Region**		
Urban	119 (16.1)	25 (10.1)
Rural	138 (10.3)	30 (7.0)
**Sputum smear**		
Negative	72 (10.4)	21 (8.0)
Positive	160 (12.7)	27 (7.2)
Unknown	25 (19.8)	7 (21.1)
**Age**		
< 51	134 (15.6)	34 (10.1)
>50	123 (10.2)	21 (6.2)
**Test year**		
2019.6–2020.5	100 (13.2)	25 (11.8)
2020.6–2021.5	80 (13.6)	13 (7.2)
2021.6–2022.5	77 (10.6)	17 (6.0)

**Figure 2:**
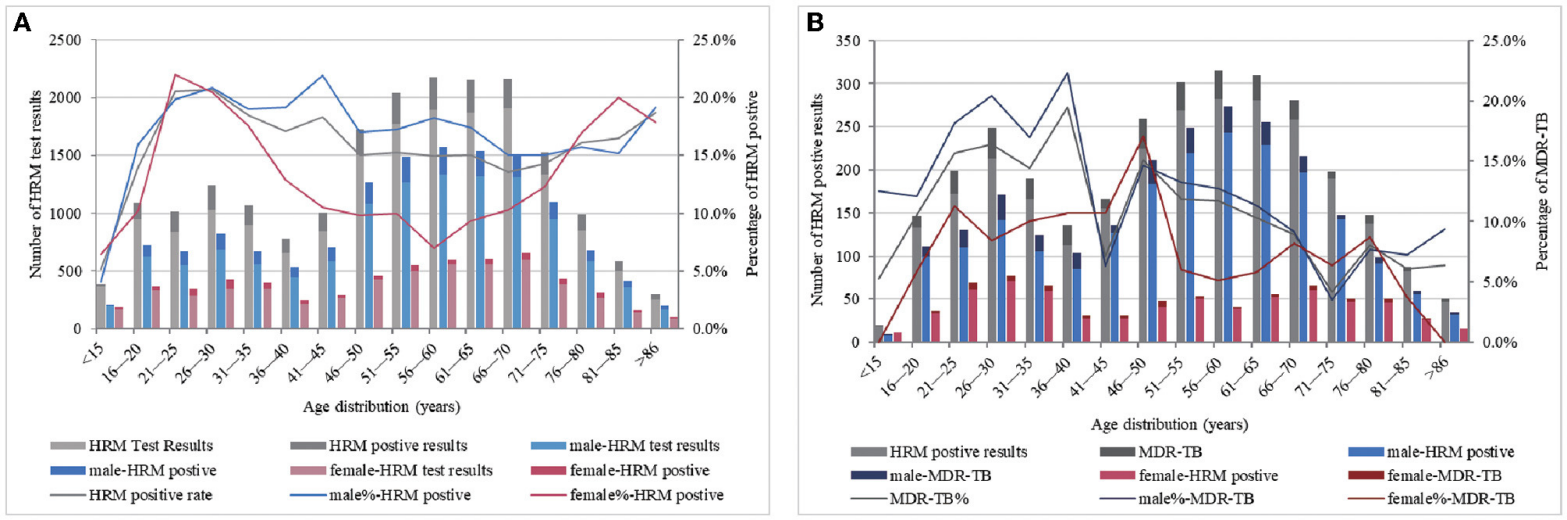
MTB-positive results **(A)** and the detection of MDR-TB **(B)** by age and gender category. **(A, B)** ordinate represent the total number of participating HRM test results and the total number of HRM positive results, respectively, and abscissa represents the age distribution. Based on the number of the HRM test, positive and MDR-TB detection by age category are presented as columnar bars, and their proportions are presented as broken lines (second y-axis).

When stratified by age, the highest positive peak rate of HRM in males was observed at 41–45 years of age, reaching 21.9%, after which it gradually decreased. Across all age groups, the positive rate remained at more than 15.0%. In contrast, the females had their highest HRM positive rate peak at a younger age, between 21 and 25 years (22.0%), which then dropped rapidly, reaching 7.0% for the 56–60 year age group. The positive rate then began to increase again, reaching 20.0% in the 81–85 year age group (Figure 2A). Overall, the males had a higher rate of MDR detection than the females across different age groups. The highest MDR-TB detection rate in females reached 17.1% at 46–50 years of age, while the peak in males occurred at 36–40 years of age (22.4%) but decreased rapidly in the two age ranges of 41–45 and 71–75 years, falling to 6.3% and 3.5%, respectively, and females showed a higher level than males (Figure 2B). To this end, various age groups of HRM-positive individuals were studied, and it was found that the prevalence of *Mycobacterium tuberculosis* (MTB) infection in urban males aged 41–45 and 71–75 years (33.9 and 28.0%) was less than the average ratio in urban males of all ages (37.1%), while that in rural males aged 41–45 and 71–75 years had a higher proportion (66.1 and 72.0%) than the average rate for all age groups of rural males by 62.9%.

**Figure 3:**
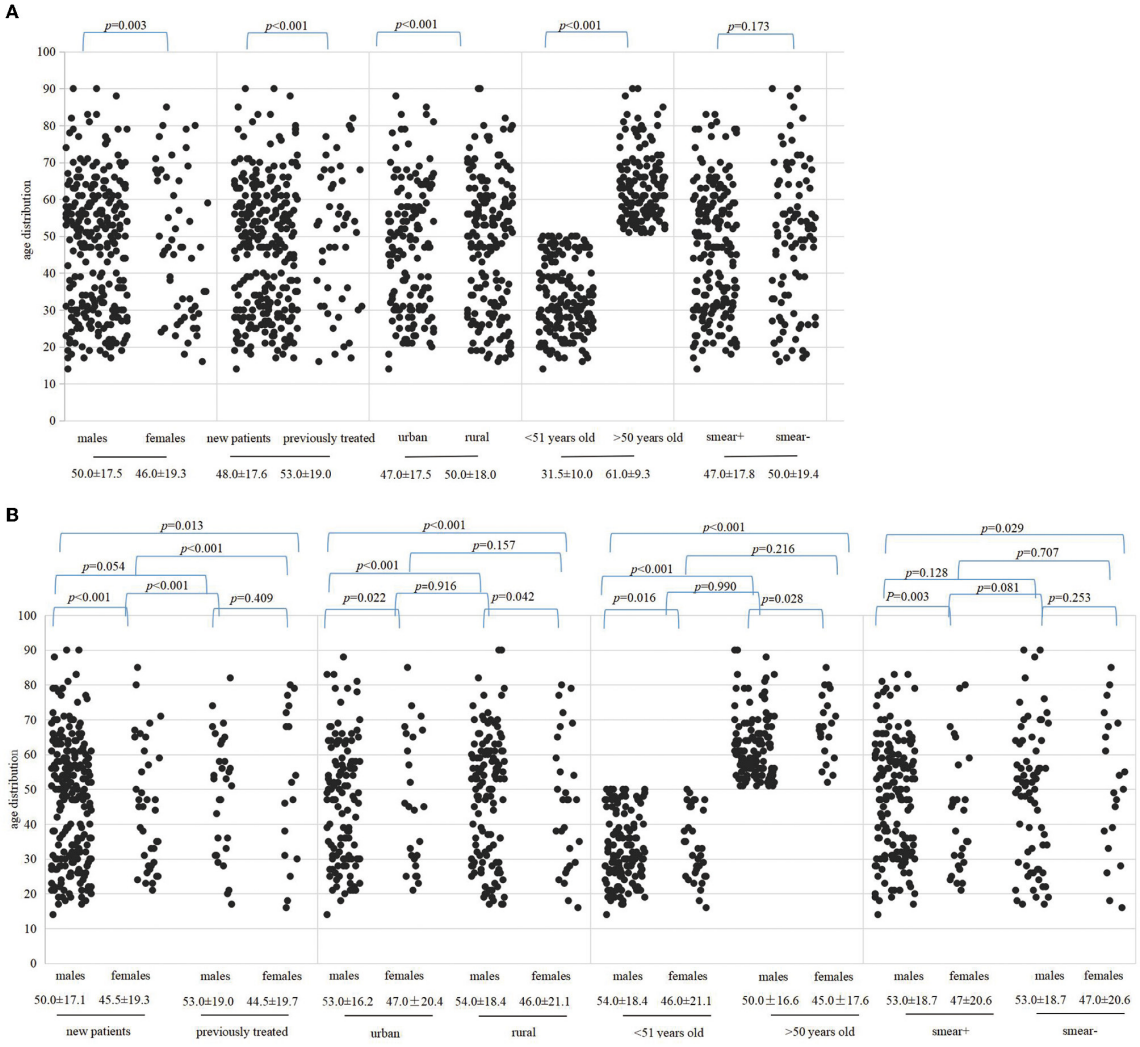
Represented correlations among patient age, gender, treatment history, region, and sputum smear by scatter plots. The dots indicate the age of patients of MDR and the median age is presented below the variables. **(A)** Indicates the difference in MDR in the context of different variables. **(B)** Indicates differences in the detection of MDR between genders under different variables. Variables include gender category, TB history, areas, older than 50 years or younger than 51 years and sputum smear results, respectively.

The Pearson's chi-squared analysis was performed to determine whether the difference in the detection of MDR-TB was statistically significant under various factors, and then, we plotted age distribution scatter plots using age categories as the ordinate and different variables as the abscissa (Figure 3). Overall, the difference in the detection rate of MDR-TB among the population infected with MTB was reflected in the male and female patients (*p* = 0.003), between cases with and without TB treatment (*p* < 0.001), between urban and rural groups (*p* < 0.001), and between groups younger than 51 years and older than 50 years (*p* < 0.001). Although the MDR detection rate of the sputum-positive group was higher than that of patients with negative smearing, the difference was not statistically significant (*p* = 0.173), as illustrated in Figure 3A. There was a statistically significant difference in MDR detection between newly diagnosed males and females (*p* < 0.001) but not between retreatment males and females (*p* = 0.409). The statistical difference detected in MDR was between urban females and males (*p* = 0.022), between rural males and females (*p* = 0.042), and between urban males and rural females (*p* < 0.001). However, between urban females and rural males and between rural females and urban females, there were no significant differences (*p* = 0.916, *p* = 0.157). Different age groups showed different levels of drug resistance. A statistically significant difference in the detection rate of MDR was reflected in males and females under 51 years of age (*p* = 0.016), males and females over 50 years of age (*p* = 0.028), as well as between females older than 50 years and males younger than 51 years (*p* < 0.001). Moreover, a statistically significant difference was observed in the detection of MDR-TB between males and females with positive sputum smearing (*p* = 0.003), as well as between females with negative smears and between males with positive smears (*p* = 0.029). Please refer to Figure 3B.

**Figure 4:**
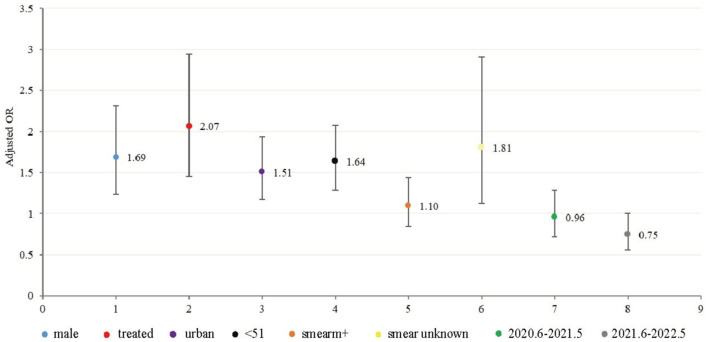
Graphic of the model of multivariate logistic regression for MDR -TB (312 MDR out of 2748 HRM positive cases from June 2019 to May 2022). The OR values and 95% confidence intervals were shown on the graph.

A multivariate logistic regression analysis revealed that the males were 1.69 (95% CI 1.24, 2.31) times more likely than the females to be diagnosed with MDR-TB. Patients with previous TB treatment were 2.07 (95% CI: 1.46, 2.94) times more likely to be diagnosed with MCR-TB than in initial cases. Cases of MDR in urban areas are 1.51 times more likely than those in rural areas (95% CI: 1.17, 1.94). Furthermore, individuals under 51 years of age had a 1.64-fold higher likelihood of being diagnosed with MDR-Tb than those over 50 years old (95% CI: 1.29, 2.08) (Figure 4).

**Figure 5:**
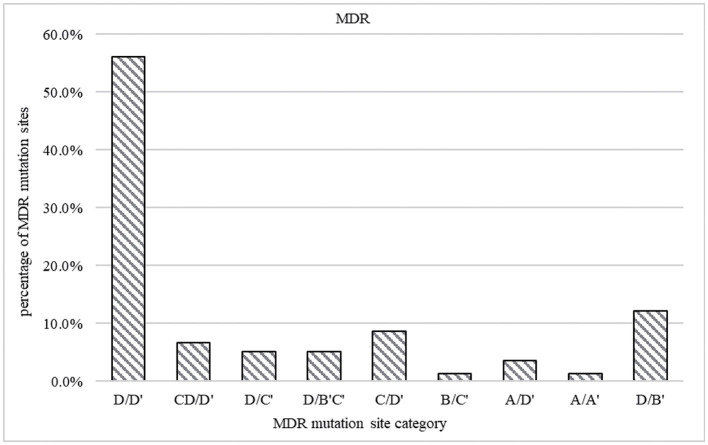
Prevalence of mutations in MDR strains. The gene coverage region and mutation points are A *ahpC* promoter region (−44 ~ −30 and −15 ~ 3 sites), B codon inhA94, C *inhA* promoter region (−17 ~ −8 site), and D codon *KatG* 315. A' codon 507–512, B' codon 521–528, C' codon 513–520, and D' codon 529–533.

The most common patterns of locus mutation in MDR were codon *KatG* 315 combination with codon 529–533, codon *KatG* 315 combination with codon 521–528, *inhA* promoter region (−17 ~ −8 sites) combination with codon 529–533, *inhA* promoter region (−17 ~ −8 site) and codon *KatG* 315 combination with codon 529–533, codon *KatG* 315 combination with codon 513–520, AhpC promoter region (−44 ~ −30 and −15 ~ 3 sites) combination with codon 529–533, AhpC promoter region (−44 ~ −30 and −15 ~ 3 sites) combination with codon 507–512, and codon inhA94 combination with codon 513–520, accounted for 56.1, 12.2, 8.7%, 6.7, 5.1, 5.1, 3.5, 1.3, and 1.3%, respectively (Figure 5).

## Discussion

The MDR detection rates showed a decreasing trend between June 2019 and May 2022, which indicates that efforts against TB have been gaining more attention in recent years. Management of DR-TB has yielded some positive outcomes and is moving in the right direction. However, it is worth noting that a global public outbreak, the COVID-19 pandemic, occurred during the study period, which has significantly hindered the positive trend of tuberculosis prevention and control (13). According to the WHO, the number of notified TB cases decreased by 18.0% in 2020 compared to 2019 (14). This was largely due to the reduction in the number of TB cases diagnosed and limited access to TB healthcare services due to the strict COVID-19 prevention and control measures and changes in the disease itself. However, the COVID-19 pandemic has masked the positive impact of measures such as mask use and social distancing, which could have potentially reduced the transmission of MTB. As a result, the burden of tuberculosis and associated mortality has increased (15). Since the SARS-CoV-2 outbreak in 2003, strict and comprehensive prevention and control measures have been implemented in the local area for the first time. These measures are likely to affect the prevalence and outcome of DR-TB, indicating that there is still a long way to go in managing local MDR-TB. The specific impact of the COVID-19 pandemic on local DR-TB will be further explored in subsequent studies.

The gender distribution characteristics of the TB-infected population conformed to the expected pattern (2), where males are more susceptible to MDR-TB. This may be due to their lifestyle and habits (16). MTB infection was found to be 3.1 times higher in males than females, while males were detected with MDR-TB at a rate of 4.7 times higher. These findings suggest that males bear a higher burden of MDR-TB and that MDR-TB is not solely due to a higher incidence of tuberculosis in males, which is consistent with previous reports (17). In addition, the MDR detection rate was significantly higher in the retreatment group than in the initial group. This indicates that treatment history plays a crucially positive role in the development of acquired resistance.

The carrier rate of multidrug-resistant *Mycobacterium tuberculosis* in the local primary tuberculosis populations (10.6%) was higher than the national average of 7.1%, indicating that there is a need to further reduce drug resistance in the primary treatment group by strengthening the management of tuberculosis patients. Although the resistance rate in the retreatment population (18.3%) was lower than the national level of 24.0% (18), the resistance rate in the retreatment group has remained high in the past 3 years (18.1% −19.4% −17.8%, data not shown), indicating an urgent need to standardize clinical medication. Additionally, the development of more precise and rapid methods for detecting tuberculosis susceptibility in developing countries is crucial for implementing clinically accurate anti-tuberculosis therapy, thereby reducing the additional burden of acquired drug resistance due to empirical medication.

Rural areas continue to be the hardest hit by tuberculosis infection. However, drug-resistant tuberculosis is more serious in urban areas. MTB is known to spread easily in high-density populations, and urban populations are relatively dense and mobile, making it easier for MTB to spread (19). Furthermore, the excellent medical infrastructure in cities leads to the accumulation of complex, difficult, or terminal tuberculosis cases, which contributes to the higher detection rate of drug-resistant tuberculosis in urban areas.

The difference in MDR detection rates between smear-positive and smear-negative cases was not statistically significant. In addition, a multivariate regression model showed an R value of 1.1 for smear-positive individuals, indicating an increased risk of MDR. Therefore, further investigation is necessary to determine whether smear positivity is associated with MDR.

Unexpectedly, MDR detection was found to occur in younger age groups for both males and females (Figure 2), which might be associated with poor medication adherence among adolescents and a greater likelihood of having been to crowded places such as schools and the army (20). The higher rates of tuberculosis resistance in young adults also present potential opportunities for screening for drug-resistant TB in groups that are traditionally considered low-risk (21). Over a lifetime, the overall rate of drug resistance was higher among males than females. However, females aged 21–25 are a group that should be focused on. An analysis of the population characteristics of males aged 41–45 years with a low detection rate of MDR revealed that rural males in this group were relatively high, and the multivariate regression analysis found that living in rural areas was a protective factor for MDR. This might be one reason for the emergence of low TB resistance in this age group.

In the multivariate model of our study, male sex, age under 51 years, urban location, and treatment history were all found to be risk factors for MDR-TB (22–24). However, some scholars have shown that females (25) and older adults (26) are also at an increased risk of TB drug resistance, indicating that the spread of DR-TB is complex and diverse. These findings also highlight the challenges of managing and controlling TB in China.

Although resistance to rifampicin and isoniazid is significantly associated with mutations in the *Mycobacterium tuberculosis* resistance genes *rpoB, katG, inhA*, and *ahpC*, these mutations vary in location and frequency in different regions (27, 28). For example, the mutation rate at *rpoB* 531 is as high as 80.9% in Kazakhstan and only 31% in Hungary, and the two mutation patterns of *katG* 315 and *inhA* account for a total of 79.9% in Beijing, China (27). The *ahpC* gene mutation, a marker of damage to the katG gene (28), was found to be 19.0% in Qinghai Province, China, while it was found to be only 4.8% in this study, which was close to the rate of 5.0% found in Poland (29). Therefore, monitoring mutation patterns at these hotspot sites is important for the timely detection of clinical TB resistance. Moreover, the most important genetic mutations associated with the spread of MDR in this region were mainly combined alterations at codons *KatG* 315 and codons 529–533 (located in the *ropB* gene), which is similar to previous reports (30, 31). Understanding the mutational mechanisms of these two codons in resistance and blocking them is crucial to preventing the spread of MDR-TB.

## Conclusion

This study aimed to identify group characteristics and risk factors associated with the transmission of MDR-TB in confirmed and suspected TB cases to provide epidemiological data for local tuberculosis prevention and control efforts. These data suggest that targeted testing for MTB infection is warranted in males aged 41–45 years and females aged 21–25 years. Moreover, patients younger than 51 years, retreatment cases, and urban TB-infected patients should be targeted for intensive surveillance of MDR-TB management. Focused detection of mutations at gene *ropB* (codons 529–533) and *KatG* 315 can facilitate early intervention in MDR-TB cases.

## Data availability statement

The original contributions presented in the study are included in the article/supplementary material, further inquiries can be directed to the corresponding authors.

## Ethics statement

Ethical review and approval was not required for the study of human participants in accordance with the local legislation and institutional requirements. Written informed consent from the participants was not required to participate in this study in accordance with the national legislation and the institutional requirements.

## Author contributions

YX and ZZ provided funding support and conceived the idea. ZW was responsible for the whole article's conception, writing, and data analysis. YH, TG, and TJ data collation, analysis, and graph construction. LX and HH revised and reviewed the manuscript. All authors contributed to the article and approved the submitted version.
